# Toxic Compound, Anti-Nutritional Factors and Functional Properties of Protein Isolated from Detoxified *Jatropha curcas* Seed Cake

**DOI:** 10.3390/ijms12010066

**Published:** 2010-12-28

**Authors:** Donlaporn Saetae, Worapot Suntornsuk

**Affiliations:** Department of Microbiology, Faculty of Science, King Mongkut’s University of Technology Thonburi, Bangkok 10140, Thailand; E-Mail: sdonlaporn@yahoo.com

**Keywords:** Jatropha curcas, protein isolate, functional properties, anti-nutritional factors

## Abstract

*Jatropha curcas* is a multipurpose tree, which has potential as an alternative source for biodiesel. All of its parts can also be used for human food, animal feed, fertilizer, fuel and traditional medicine. *J. curcas* seed cake is a low-value by-product obtained from biodiesel production. The seed cake, however, has a high amount of protein, with the presence of a main toxic compound: phorbol esters as well as anti-nutritional factors: trypsin inhibitors, phytic acid, lectin and saponin. The objective of this work was to detoxify *J. curcas* seed cake and study the toxin, anti-nutritional factors and also functional properties of the protein isolated from the detoxified seed cake. The yield of protein isolate was approximately 70.9%. The protein isolate was obtained without a detectable level of phorbol esters. The solubility of the protein isolate was maximal at pH 12.0 and minimal at pH 4.0. The water and oil binding capacities of the protein isolate were 1.76 g water/g protein and 1.07 mL oil/g protein, respectively. The foam capacity and stability, including emulsion activity and stability of protein isolate, had higher values in a range of basic pHs, while foam and emulsion stabilities decreased with increasing time. The results suggest that the detoxified *J. curcas* seed cake has potential to be exploited as a novel source of functional protein for food applications.

## 1. Introduction

*Jatropha curcas* is a multipurpose plant which belongs to the *Euphorbiaceae* family. It is thought to be native to Central and South America and widely distributed in Central America, Africa and Asia. Recently, it has been widely planted in Thailand and promoted as a biodiesel plant. Its seed contains 60–66% crude lipid and 30–32% crude protein [1]. Increasing *J. curcas* production as a biofuel source will increase the quantity of its seed cake, which is a by-product. The seed cake left after extraction of oil provides a high amount of protein [2] and all concentrations of essential amino acids except lysine are higher than those of the Food and Agriculture Organization (FAO) reference pattern suggested for pre-school children [3]. However, major constituents contained in the seed cake are toxic compound and anti-nutritional factors. The main toxic component of the seed cake is phorbol esters and the anti-nutritional factors found in the seed cake are trypsin inhibitor, phytic acid, lectin and saponin.

Phorbol esters have been classified as the main toxic agents in *J. curcas* seed cake responsible for toxicity [4]. The toxicity of phorbol esters limits the use of *J. curcas* seed cake as a human or animal food. The phorbol esters affect humans and animals by causing tumor promotion, cell proliferation, blood platelet activation, lymphocyte mitogenesis, erythema of the skin, prostaglandin production and stimulation of degranulation in neutrophils [5]. If the phorbol esters were removed, the *J. curcas* seed cake could be a protein-rich ingredient in food or feed diets and would also provide a valuable protein.

Plant protein isolates are important ingredients for protein sources in human and animal feed. The protein isolation generally consists of two steps, which are protein solubilization in alkaline solution and protein precipitation. For plant protein precipitation, acid precipitation at the isoelectric pH is the general method used. Since plant protein isolates are less expensive sources than animal ones, they are used to fortify, formulate and apply food products with desirable functional properties. Important functional properties required in protein ingredients include solubility, water and oil binding capacities, emulsion activity and stability and also foam capacity and stability. The present study focused on the toxin, anti-nutritional factors and functional properties of protein isolated from detoxified *J. curcas* seed cake.

## 2. Results and Discussion

### 2.1. Chemical Compositions

The chemical composition of *J. curcas* seed cake, detoxified seed cake and protein isolated from the detoxified seed cake are shown in Table 1. The protein content of both *J. curcas* seed cake and detoxified seed cake were similar, approximately 23%, suggesting that the ethanol extraction did not affect the protein content; the protein contents were also similar to that reported by Makkar *et al.* [6]. However, the other chemical compositions of both seed cakes, especially fat and fiber, were considerably different, as indicated in Table 1, since ethanol extraction heavily affected fat and fiber. The yield of proteins isolated from the detoxified seed cake was approximately 70.9%, which is similar to that reported by Devappa and Swamylingappa [7]. The protein content of the protein isolate (89.0%) was approximately four-fold higher than that of the detoxified seed cake. Color of the protein isolate was found to be brownish, partly resulting from the dark color of the *J. curcas* seed shells. Its color characteristic agreed well with the reports of Saetae *et al.* [8] and Devappa and Swamylingappa [7].

### 2.2. Toxic Compound and Anti-Nutritional Factors

The contents of toxin and anti-nutritional factors detected in *J. curcas* seed cake, detoxified *J. curcas* seed cake and its protein isolate are presented in Table 2. The results show that the phorbol ester and lectin contents were not detected in the detoxified *J. curcas* seed cake, whereas they were observed in high levels in *J. curcas* seed cake. Phytic acid, trypsin inhibitor and saponin found in the detoxified seed cake were also much lower than those found in *J. curcas* seed cake. This suggests that ethanol extraction could be an effective method to completely remove the phorbol esters and lectin and partially remove the phytic acid, trypsin inhibitor and saponin from the seed cake.

No phorbol esters and lectin were found in the protein isolated from the detoxified seed cake (Table 2). The phytic acid detected in the protein isolate was 0.03%, which was much lower than that of the detoxified seed cake. This result implies that the phytic acid content in the protein isolate was reduced by 98% from the seed cake. This might be due to the protein isolation process resulting in binding of phytate ions to sodium ions in alkali solution. Similar observations were reported in several works [7,9,10]. The trypsin inhibitor level of the protein isolate was approximately eight-fold higher than that of the detoxified seed cake. Makkar *et al.* [6] also reported that the trypsin inhibitor detected in *J. curcas* protein concentrate was approximately 10-fold higher than that of the seed cake. This result indicates that the trypsin inhibitor was solubilized and precipitated at the same pH as other proteins so that it was found in high level in the protein isolate. The saponin content of the protein isolate was found in a very low level. It was decreased by approximately 80% from the seed cake. This suggests that the saponin was partially removed by protein isolation as mentioned in several reports [7,9,10].

### 2.3. Functional Properties of Protein Isolate

#### 2.3.1. Solubility

**Figure 1:**
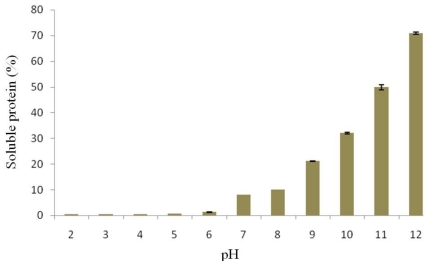
Effect of pH on the solubility of the protein isolated from detoxified *J. curcas* seed cake.

The solubility of proteins isolated from the detoxified seed cake as a function of pH in a range of 2.0 to 12.0 is illustrated in Figure 1. High solubility of protein is an important characteristic and is required for protein isolates to be applied as functional ingredients in foods [11]. The pH is an important factor since it affects the protein solubility. The solubility of the protein isolate observed in this study followed a U-shape curve, which is typically found in general legume seed proteins. The solubility of the protein isolate was low at acidic pH condition and increased with increasing pH values. The solubility was minimal at pH 4.0, indicating its isoelectric point. Similar observations were reported by Makkar *et al.* [6], Saetae *et al*. [8], Vani and Zayas [12] and Mohamed *et al.* [13]. At the isoelectric point, the net charge of protein molecules is zero and the protein itself is precipitated. The maximum solubility of protein isolate was reached at pH 12.0. The results suggest that the protein isolate had good solubility under basic conditions.

#### 2.3.2. Water and Oil Binding Capacities

Water binding capacity of proteins is important in viscous foods in order to provide body, thickening and viscosity. The water binding capacity of the protein isolated from the detoxified seed cake (1.76 g water/g protein) was similar to that reported by several studies [14–18], which indicate that this protein isolate had good water binding capacity possibly due to the interactions between polar amino acid residues of protein and water molecules. However, its water binding capacity was much lower than that of protein isolated from the seed cake without detoxification [8], since ethanol extraction might disrupt the internal structure and change the protein conformation, surface polarity and surface hydrophobicity of the protein in the seed cake.

Oil binding capacity is another important functional property of proteins in food systems. It is a critical property of flavor retention. The oil binding capacity of the protein isolated from the detoxified seed cake was found to be 1.07 mL oil/g protein. It was lower than that of the protein isolated from the seed cake without detoxification (1.86 mL oil/g protein) [8], cowpea protein isolates (2.0–2.22 mL oil/g protein) [19] and soy protein isolate (3.29 mL oil/g protein) [20]. The result suggests that the protein isolate had low non-polar amino acid contents to bind with hydrocarbon chains of lipids.

#### 2.3.3. Foam Capacity and Stability

**Figure 2:**
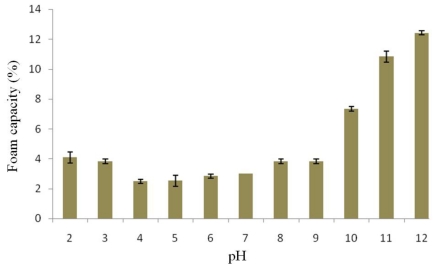
Effect of pH on foam capacity of protein isolated from detoxified *J. curcas* seed cake.

Factors affecting foam capacity and stability of proteins are the type of protein, degree of denaturation, pH, temperature and whipping methods. Foam can be produced by whipping air into liquid quickly as much as possible. The foam capacity of protein isolated from the detoxified seed cake was pH-dependent, as presented in Figure 2. The protein isolate showed low foam capacity values at a pH range of 4.0–6.0 and the foam capacity was maximized at pH 12.0. The foam capacity values increased under basic pH conditions. The higher capacity values are due to an increase in protein solubility where soluble proteins can reduce surface tension at the interface between air bubbles and the surrounding liquid. The foam capacity of this protein isolate was similar to that of the protein isolated from the seed cake without detoxification [8] but was lower than that of proteins isolated from other seeds [18,21]. This result indicates that the major protein molecules of *J. curcas* may be globular proteins since they were difficult to denature at the surface of their molecules leading to low foam capacity values [22].

**Figure 3:**
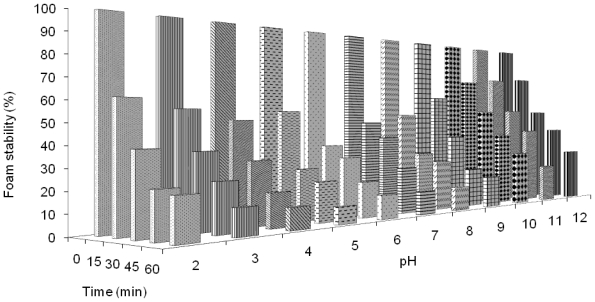
Effect of pH and time on foam stability of protein isolated from detoxified *J. curcas* seed cake.

The effect of time and pH on the foam stability of the protein isolated from the detoxified seed cake is shown in Figure 3. The foam stability decreased with increasing time ranging from 15 to 60 min. El-Nasri and El-Tinay [16] also reported a similar observation. The foam stability markedly decreased within the first 15 min and slightly decreased after 30 min. The minimum stability of the protein isolate at the first 15 min was in a pH range of 4.0 to 6.0 (37–56%). This result suggests that the interaction of protein-protein molecules at the isoelectric point leads to low foam stability values. The foam stability increased above and below the isoelectric pH. The maximum foam stability of the protein isolate at the first 15 min was observed in a pH range of 9.0 to 12.0 (65–80%). It was due to increasing protein solubility. After 60 min, the low foam stability was observed in a pH range of 4.0 to 6.0 (9–12%) and high stability was found between pH values of 10.0 to 12.0 (22–30%). The overall foam stability of this protein was similar to that of the protein isolated from the seed cake without detoxification [8].

#### 2.3.4. Emulsion Activity and Stability

**Figure 4:**
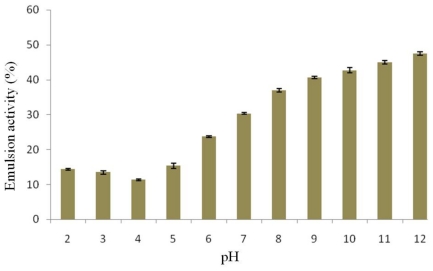
Effect of pH on emulsion activity of protein isolated from detoxified *J. curcas* seed cake.

Proteins generally possess hydrophobic and hydrophilic properties due to different types of amino acids. This causes protein interaction with both oil and water molecules and the proteins can act as emulsifiers. Emulsion capacity depends on the hydrophobic-hydrophilic balance, which is affected by pH. The emulsion activity of protein isolated from detoxified *J. curcas* seed cake was pH-dependent as shown in Figure 4. The emulsion activity was low under acidic pH conditions, because proteins were low in solubility and lacked electrostatic repulsive forces which led to decreased emulsion formation. The emulsion activity of the protein isolate was found to be low in a pH range of 3.0–5.0. Its value increased with increasing pH and was maximal at pH 12.0 due to the highest protein solubility. The emulsion activities of this protein at each pH were lower than those of the protein isolated from the seed cake without detoxification [8], which agreed well with their water and oil binding capacities. In addition, the emulsion activity of detoxified *J. curcas* seed cake at pH 12.0 was slightly different from that of fenugreek protein concentrate [16].

**Figure 5:**
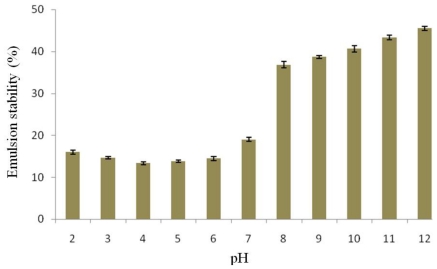
Effect of pH on emulsion stability of protein isolated from detoxified *J. curcas* seed cake.

A suspension of protein isolate is generally heated in order to study its emulsion stability. This step leads to protein unfolding and the hydrophobic residues located inside the protein structure being exposed. This causes better adsorption of the protein molecules on an oil-water interface. The emulsion stability of protein isolated from the detoxified *J. curcas* seed cake was pH dependent (Figure 5). The emulsion stability was low under acidic pH conditions. The low emulsion stability at the isoelectric pH might be attributed to lack of electrostatic repulsive interactions among particles, which promotes flocculation and coalescence [23]. The emulsion stability of protein isolate was greatly different under basic pH conditions (8.0–12.0) and it was highest at pH 12.0. The emulsion stabilities of this protein at each pH were still lower than those of the protein isolated from the seed cake without detoxification [8], resulting from the change of protein conformation, surface polarity and surface hydrophobicity caused by ethanol extraction.

## 3. Experimental Section

### 3.1. Material

*J. curcas* seed cake was kindly provided from Ladda Company Limited. The whole seeds were pressed by a screw press and the oil was removed to obtain the seed cake. The seed cake was ground and packaged in polyester plastic bags. It was stored in a freezer at −20 °C prior to use.

### 3.2. Preparation of Detoxified *J. curcas* Seed Cake

*J. curcas* seed cake was detoxified by ethanol extraction [24] in order to remove phorbol esters. Five grams of the seed cake was extracted by 15 mL of 90% (v/v) ethanol with a shaking speed of 150 rpm for 5 min at room temperature. This extraction was repeated four times. The seed cake passed through this process was referred to as the detoxified *J. curcas* seed cake.

### 3.3. Preparation of Protein Isolates

The detoxified *J. curcas* seed cake was suspended in distilled water (1:10). The pH of suspension was adjusted to 12.0 with 1 N NaOH and stirred at 50 °C for 3 h. Then, the suspension was centrifuged at 1837 × g (Rotanta 46R, Hettich, Germany) for 30 min. The supernatant was collected and adjusted to pH 4.0 with 1 N HCl for protein precipitation. The precipitated proteins were collected by centrifugation and washed twice by distilled water and then dried by vacuum at 40 °C.

### 3.4. Chemical Compositions

Crude protein, crude lipid, crude fiber and crude ash contents of *J. curcas* seed cake, detoxified *J. curcas* seed cake and its protein isolate were determined according to the standard methods of the Association of Official Analytical Chemists (AOAC) [25]. Their available carbohydrate contents were calculated by difference. All analyses were performed in triplicate.

### 3.5. Determination of Toxic Compound and Anti-Nutritional Factors

Toxic compound and anti-nutritional factors of *J. curcas* seed cake, detoxified *J. curcas* seed cake and its protein isolate were analyzed.

Phorbol esters were extracted by the method of Saetae and Suntornsuk [24] and analyzed by HPLC according to the modified method of Hass and Mittelbach [26]. The analytical column was a Nova-Pak C18 column (150 × 3.9 mm i.d. and 4 μm particle size, Waters), with a SB-C18 guard column (12.5 × 4.6 mm i.d. and 5 μm particle size) (Agilent). The column was thermally controlled at 25 °C. A mixture of acetonitrile (HPLC grade, Fisher Scientific, Leicestershire, UK) and deionized water (80:20, v:v) was used as the mobile phase at a flow rate of 1 mL/min. The photodiode detector wavelength was set at 254 nm. The phorbol 12-myristate 13-acetate (PMA) (Sigma Chemical) was used as an external standard.

Phytic acid was determined by a colorimetric method described by Vaintraub and Lapteva [27]. Results were expressed as % (w/w) using phytic acid (sodium salt, Sigma Chemical) as a standard.

Trypsin inhibitor activity was determined by the method of Kakeda *et al.* [28] using benzoyl-DL-arginine-*p*-nitroanilide (BAPNA) as a substrate and porcine trypsin (Type II-S, Sigma Chemical). The result was expressed as trypsin inhibitor unit (TIU) per gram sample (detoxified seed cake or protein isolate).

Lectin activity was determined by the modified method of Gordon and Marquardt [29] by using hemagglutination assay.

Saponin content was determined by a spectrophotometric method described by Thilborg *et al.* [30]. The result was expressed as diosgenin equivalent from a standard curve of different concentrations of diosgenin (Sigma Chemical) in 80% (w/v) aqueous methanol.

### 3.6. Functional Properties of Protein Isolate

Protein solubility was studied in the pH range of 2.0 to 12.0. Protein isolate (5 g) for each pH was suspended in 20 mL of distilled water. The pH of the suspension was adjusted using 0.1 N HCl or 0.1 N NaOH. The suspension was shaken for 1 h at 30 °C and centrifuged at 2000 × g for 30 min. The protein content of the supernatant was determined by the method of Bradford [31]. Water and oil binding capacities were determined by the modified method of Beuchat [32] and Chakrabokry [33], respectively. Foam capacity and stability were determined by the modified method of Makri *et al.* [34]. Emulsion activity and stability were determined by the modified method of Naczk *et al.* [35].

## 4. Conclusions

Toxins and anti-nutritional factors in *J. curcas* seed cake could be totally or partially removed by ethanol extraction to leave a detoxified *J. curcas* seed cake. Protein isolated from the detoxified *J. curcas* seed cake showed high protein content (89%). All functional properties of the protein isolate were observed to be good under neutral to basic pH conditions. Therefore, the protein isolated from the detoxified seed cake could be a good protein source for food systems since the protein contained no toxins and lectin. Studies in animal tests should be investigated to confirm the safety of protein isolated from the detoxified *J. curcas* seed cake before its applications into foods.

## Figures and Tables

**Table 1:** Chemical compositions of *J. curcas* seed cake, detoxified seed cake and its protein isolate (on dry matter basis).

Composition (%, w/w)	*J. curcas* Seed Cake 1,2	Detoxified *J. curcas* Seed Cake 1	Detoxified Seed Cake Protein Isolate 1
Crude fat	14.8 ± 0.5	8.6 ± 1.6	7.1 ± 0.4
Crude fiber	11.0 ± 1.7	8.2 ± 0.3	0.7 ± 0.1
Crude ash	7.8 ± 0.1	6.4 ± 0.2	1.8 ± 0.1
Crude protein	23.5 ± 1.5	23.0 ± 1.0	89.0 ± 1.8
Available Carbohydrate	42.9	53.8	1.4

1 Means ± standard deviation of triplicate determinations;

2 Saetae *et al.* [8].

**Table 2:** Toxic compound and anti-nutritional factors in *J. curcas* seed cake, detoxified seed cake and its protein isolate.

Component	*J. curcas* Seed Cake 1,2	Detoxified *J. curcas* Seed Cake 1	Detoxified Seed Cake Protein Isolate 1
Phorbol esters (mg/g dry sample) a	0.73 ± 0.06	ND 3	ND 3
Phytic acid (%, w/w)	8.55 ± 0.51	1.87 ± 0.11	0.03 ± 0.00
Trypsin inhibitor (TIU b/g dry sample)	7.42 ± 1.64	1.12 ± 0.09	8.36 ± 0.03
Lectin activity (HU c/mg protein)	13.15 ± 0.45	ND 3	ND 3
Saponin d (μg/g dry sample)	27.82 ± 0.68	10.04 ± 0.60	2.04 ± 0.01

1 Means ± standard deviation of triplicate determinations;

2 Saetae *et al.* [8];

3 ND: not detected;

a Equivalent to phorbol 12-myristate 13-acetate;

b Trypsin inhibitor units;

c Hemagglutinating units;

d Diosgenin equivalents.
